# Preschool children’s preferences for sedentary activity relates to parent’s restrictive rules around active outdoor play

**DOI:** 10.1186/s12889-019-7235-x

**Published:** 2019-07-15

**Authors:** Nicola Wiseman, Neil Harris, Martin Downes

**Affiliations:** 1 School of Medicine, Griffith University, Gold Coast Campus, Gold Coast, Australia; 2 Centre for Applied Health Economics, Menzies Health Institute Queensland, School of Medicine, Griffith University, Nathan campus, Gold Coast, QLD 4111 Australia

**Keywords:** Physical activity, Preschool children, Parenting practices, Screen-time

## Abstract

**Background:**

With prevalence estimates indicating that young Australian children are increasingly sedentary, it is important to identify the relevant attributes that are shaping this lack of activity. Literature has identified safety concerns of parents as a consistent barrier to physical activity participation of young children. Despite safety being a plausible determinant of young children’s activity preferences, the impact of restrictive parenting practices has rarely been examined through quantitative research. The current study investigates the link between controlling and supportive physical activity parenting practices and preschool children’s physical activity knowledge, preferences and parent-reported behaviour.

**Methods:**

The current cross-sectional study included 138 parent-child dyads and involved two components of data collection including a child and a parent questionnaire. Results of the parent and child questionnaires were matched to determine correlations between physical activity parenting practices and preschool children’s physical activity knowledge, preference and parent-reported behaviour.

**Results:**

Children’s preferences for physical activity correlated with a number of demographic characteristics and physical activity parenting practices, with the most influential variables being parental age, parental rules around active play outdoors and parental use of screen-time to reward/control child behaviour. Based on parental-reporting, children who preferred to be physically active were more likely to engage in physical activity and were less likely to engage in screen-time on the weekend.

**Conclusions:**

This study identified that parenting practices are not only associated with children’s active and sedentary behaviours (parent-reported), but also with how children prefer to play (parent-reported). Future research should seek to clarify the relationship between children’s activity preferences and parent’s use of screen-time to reward and control their child’s behaviour, given the developmental and behavioural health risks associated with excessive media/screen exposure in early childhood. Further research should investigate whether competing societal values of the importance of encouraging children’s risky play and the need to prevent children from being injured, coupled with parent’s busy schedules are contributing to parental ambivalence regarding how to promote active play for their children. Finally, research should be conducted to establish the relationship between physical activity parenting practices and children’s objectively-measured activity level.

## Background

Physical activity (PA) is essential to the physical, psychological, social, and cognitive health of children [1]. Despite the well-documented benefits of physical activity for young children, evidence suggests that young children are relatively inactive. In Australia, prevalence estimates suggest that preschool-aged children engage in low levels of physical activity and are sedentary for a large proportion of their day, with watching television (including DVDs and movies) consuming more of children’s leisure time than other identified recreational activities [2, 3]. Growing evidence indicates that sedentary and active behaviours once established in early childhood, continue into adolescence, contributing to long term health outcomes [1, 4]. In response to this, a range of interventions have been implemented for preschool children to increase activity levels and decrease sedentary time before physical activity behaviours are cemented. Despite these efforts, few studies have reported having positive and lasting effects on physical activity [5]. The reported reasons for the lack of intervention success are varied, however, it has been suggested that to increase intervention success it is important to establish the relevant influences on the target behaviour [3].

Preschool children’s physical activity behaviours are the product of the interaction between individual characteristics and their environment [6]. At this age preschool children are very reliant on others for many of their behavioural choices and have limited autonomy, thus, parents are critical in influencing children’s physical activity attitudes and behaviours [7]. Unsurprisingly, behaviours of parents to encourage or limit their child’s physical activity or inactivity can influence children’s play behaviours (e.g. personal activity patterns, monitoring of child TV viewing/PA, family TV viewing) [8]. One key factor which has been recognised to contribute to children’s declining physical activity levels across many modern Western societies is the pervasiveness of ‘surplus safety’ [9–11]. This has resulted in parents restricting children’s play and limiting children’s risk of engaging in dangerous behaviours.

A recent systematic review conducted by Hesketh and colleagues (2017) of factors which facilitate or hinder children’s physical activity, identified safety concerns of parents as a central and consistent barrier to physical activity participation of young children. A number of studies in the review reported that parents were worried that a physically active child could hurt themselves and may therefore be likely to limit their child’s activity [3, 12–14]. Further, children reported that adults’ fears in relation to their safety and their health limited their activity levels [15]. Despite safety being a consistent and plausible determinant of young children’s activity behaviours, the impact of restrictive parenting behaviours has rarely been explored in the quantitative literature [3, 5].

As practices parents use to control physical activity can shape preschool children’s participation behaviours, it can be hypothesised that this parental concern for child safety may extend influence over children’s understanding of the importance of physical activity and children’s activity preference. There is much qualitative research to support that a child’s preference for more sedentary behaviours can have a negative impact on their participation in active play [16–19]. Further, how a child regards the importance and benefit of physical activity is said to play a role in determining their play choices, as they grow older and gain more autonomy over their behaviour [20]. Currently, little research has quantitatively examined the relationship between controlling and supportive physical activity parenting practices, child physical activity knowledge and preference.

This study contributes to the literature by examining the link between preschool children’s knowledge and preference for physical activity matched with a parental survey which measured controlling and supportive physical activity parenting practices. Physical activity parenting practices refers to the behaviours or actions (intentional or unintentional) performed by parents for child rearing purposes that influence their children’s attitudes, behaviours, or beliefs around physical activity [8]. The study builds on existing qualitative literature, by quantitatively examining the relationship between controlling and supportive physical activity parenting practices, children’s PA knowledge, preferences (parent and child-reported) and behaviours (parent-reported). The addition of a quantitative study to the evidence base will enhance our understanding of this relationship and may lead to more targeted interventions to address declining physical activity participation in young children.

## Methods

### Procedure

The current cross-sectional study involved two components of data collection. The first component of data collection included a parental questionnaire. The questionnaire comprised demographic questions, questions regarding their child’s physical activity/sedentary behaviours and parenting practices thought to influence children’s physical activity and inactivity (screen-time behaviours) [8]. Questions regarding screen time were modified to include more contemporary screen-time activities including iPad and smartphone. The questionnaire used was validated in a study conducted by Vaughn and colleagues (2013), and measures 14 parent practices used to either control (*n* = 6) or support (*n* = 8) children’s physical activity or screen-time [8]. Vaughn and others (2013) conducted an exploratory analysis and reported that the internal consistency for all factors within the tool was good, with Cronbach’s alphas ranging between 0.54–0.88 [8]. The questionnaire included questions regarding the physical home environment, controlling parenting practices (such as rules around indoor and outdoor active play and screen-time) e.g. How often do you ask your child to calm down his/her active play? and supportive practices including parental implicit and explicit modelling (co-activity, praise and verbal encouragement, education, enjoyment and prompting) e.g. How often does your child see you doing or going to do something that is physically active? The questionnaires were distributed throughout the participating childcare centres to parents. Upon the return of the parental questionnaire/consent form for their child’s participation, the second component of data collection was conducted.

The second component of the data collection included children being asked to complete the preschool children’s food and play questionnaire (Pre-FPQ).The pre-FPQ is a validated iPad activity that quantitatively measures preschool children’s knowledge of and preference for physical activity (https://apple.co/2Evbfwn) [21]. The Pre-FPQ was conducted by one of the researchers (NW) at participating childcare centres, in the free play area, which was visible to childcare educators and took approximately 5–10 min). Results of the parent and child questionnaires were matched to determine any correlation with parental practices related to physical activity (parent-reported) and preschool children’s knowledge and preference of physical activity (parent and child-reported), and physical activity behaviours. Children’s self-reported physical activity knowledge and preference was scored out of eight, with one point allocated for selecting the most active option out of two option photographs. Details regarding the photographs in the application and the administration of the application is available in the validation study conducted by Wiseman et al. [21]. The Griffith University Human Research Ethics Committee approved this study protocol (No: 2016/628).

### Participants

Parent-child dyads were recruited using convenience sampling at 19 childcare centres across North East New South Wales and South East Queensland, Australia in July to December of 2017. Children were required to be aged between 3 and 5 years to be included in the study. In Australia, 60% of four-year-old children attend preschool, thus this was considered an appropriate setting to access the target population [22]. The required sample size of 140 was based on previous knowledge of the expected effect size of independent variables to detect an effect size (f^2^) of 0.08 with an alpha error of 0.05 and a power of 80%.

### Data analysis

Statistical analyses were performed using Statistical Package for the Social Sciences by two of the researchers (NW and MD) (SPSS version 23.0). Descriptive statistics were used to describe the demographic characteristics of the study participants. To determine the impact of key demographic variables on independent variables, Independent samples t-tests and ANOVAs were used. Pearson correlation tests were used to examine the relationships between parenting physical activity practices and children’s physical activity knowledge, preference (parent and child-reported) and behaviours (parent-reported). Finally, multiple regression modeling was employed to examine the contribution of key physical activity parenting practices on children’s physical activity preferences (parent questionnaire), after controlling for key demographic variables. Preliminary analyses were conducted to ensure no violation of the assumptions of normality, linearity and homoscedasticity.

## Results

### Demographic characteristics of participants

A total of 162 parent child-dyads were recruited for this study, from this sample a total of 138 parent-child dyads completed the questionnaire and were included in the data analysis. Participants ranged in age from 3.1 years (37 months) to 4.9 years (59 months) (M = 49.88 months, SD = 5.42). The demographic characteristics of the sample are presented in Table 1. Approximately half of the child participants were males (66, 52%). Majority of the participating primary carers were female (122, 94.6%) and Caucasian (83%), just under two thirds of primary carers had undergone tertiary education (64.6%), and just over half had an annual household income of $90,000 or more per annum (54.1%).

**Table 1:** Descriptive statistics of demographic factors and key independent variables

Variable	
Categorical variables	n (%)
Child gender	
Male	66 (52.0)
Female	61 (48.0)
Primary carer gender	
Male	7 (5.4)
Female	122 (94.6)
Primary carer age^a^	
20–29	17 (13.2)
30–39	90 (69.0)
40^b^	22(17.0)
Ethnicity^a^	
Caucasian	113 (83.0)
Other	20 (16.9)
Marital status^a^	
Married/defacto/living together	107 (82.9)
Single/divorced/separated	22 (17.1)
Education level of primary carer^a^	
High school	28 (22.0)
Technical or trade certificate/apprenticeship	17 (13.4)
Tertiary education	82 (64.6)
Income^a^	
0 - $49,999Per annum	26 (21.3)
$50,000 – $89,999	22.1 (24.6)
$90,000–$129,999	27.9 (31.1)
$130,000–$150,000^b^	20.6 (23.0)
Continuous variables	Mean (SD)
Child age (months)	49.88 (5.42)
PA preference (Pre-FPQ)	3.83 (1.63)
PA knowledge (Pre-FPQ)	5.39 (2.03)
PA preference (Parent questionnaire)	19.6 (3.02)
Total hours child PA week^b^	11.9 (8.1)
Total hours child PA weekend^b^	7.41 (4.61)
Total hours child screen-time week^b^	6.04 (4.39)
Total hours child screen-time weekend^b^	4.21 (2.82)

^a^Categories were collapsed

^b^parent reported

On average, parents reported that children spent a total of 6.04 h during the week engaging in screen-time (total hours screen-time from Monday-Friday), and a total of 4.21 h over the weekend (Total hours screen-time from Saturday and Sunday). On average, parents reported that children spent a total 11.9 h participating in active outdoor play during the week (Total hours active outdoor play from Monday to Friday) and 7.41 h on the weekend (Total hours active outdoor play from Saturday and Sunday). Children’s average physical activity knowledge and preference scores (iPad activity) were 5.39 and 3.83 respectively (out of a possible score of 8). The higher the child’s score, the more they preferred active play (PA preference), and the more they knew about the importance of activity play (PA knowledge). The average child PA preference score reported by parents was 19.6 (out of a possible score of 25). With a higher score indicating a child’s preference for physical activity in comparison to sedentary activities.

### Demographic characteristics and children’s physical activity knowledge and preferences and behaviour

No significant relationships were found between primary carer income, gender or marital status and children’s activity participation, screen time, PA knowledge or preference by child or parent report. Analyses indicated that Caucasian children preferred active activities (child reported) (*t* = .132, *p* < 0.05) and spent more hours playing outside during the week (*t* = 3.08, *p* < 0.001) (Table 2). Child gender also appeared to be associated with activity preferences, with more males preferring PA than females. Despite this, on average, males were engaged in more weekend screen time than females (*t* = 2.00, *p* < 0.01).

**Table 2:** Children’s physical activity preference, knowledge, screen-time and outdoor play by demographic factors

Variable	Ethnicity				Child Gender				Age of Primary carer					Primary carer education				
	Caucasian	other			Male	female			20–29 years	30–39 years	40^+^ years			High School	Technical or trade certificate	Tertiary		
	Mean (SD) *n* = 109	Mean (SD) *n* = 20	*t*	p	Mean (SD) *n* = 62	Mean (SD) *n* = 60	*t*	p	Mean (SD) *n* = 17	Mean (SD) *n* = 90	Mean (SD) *n* = 22	F	p	Mean (SD) *n* = 27	Mean (SD) *n* = 16	Mean (SD) *n* = 80	F	*p*
Weekend outdoor play^a^	7.65 (4.1)	5.46 (7.5)	1.73	0.08	7.60 (4.6)	7.20 (4.7)	.379	0.71	7.46 (3.66)	7.51 (5.05)	6.95 (3.2)	.130	.878	5.50 (4.28)	6.84 (2.97)	8.15 (4.87)	3.57	0.03^***^
weekday outdoor play^a^	12.68 (8.0)	6.00 (6.1)	3.08	0.00^***^	12.1 (4.7)	12.02 (8.5)	0.31	0.97	12.9 (8.26)	11.58 (8.13)	12.6 (8.4)	.291	.748	8.64 (6.69)	10.1 (5.07)	13.52 (8.78)	4.28	0.01^**^
Weekend screen-time^a^	6.22 (4.5)	8.03 (10.5)	−0.66	0.23	4.74 (3.2)	3.71 (2.4)	2.00	0.04^*^	3.53 (1.97)	3.71 (2.47)	4.21 (2.8)	1.11	.331	4.90 (3.98)	4.43 (2.30)	4.22 (2.84)	1.20	0.30
Weekday screen-time^a^	4.10 (2.6)	5.26 (3.8)	−1.13	0.13	6.3 (4.9)	5.91 (3.8)	0.443	.658	6.59 (5.38)	6.14 (4.36)	5.21 (3.7)	0.51	.597	6.64 (5.50)	6.46 (4.17)	5.85 (4.09)	0.368	0.69
Child PA preference (Pre-FPQ)	3.92 (1.6)	3.00 (1.3)	.132	0.03^*^	4.12 (1.6)	3.35 (1.5)	2.59	0.01^**^	4.57 (1.28)	3.81 (1.64)	3.40 (1.6)	2.24	.110	4.11 (1.69)	3.80 (1.82)	3.74 (1.58)	0.500	0.60
Child PA knowledge (Pre-FPQ)	5.42 (2.0)	4.9 (1.9)	.828	0.40	5.18 2.0)	5.53 (2.0)	−.907	.367	5.00 (2.07)	5.47 (2.03)	5.27 (2.1)	.357	.70	5.29 (1.93)	5.46 (2.53)	5.37 (2.03)	0.034	0.96
Child PA preference (Parent questionnaire)	19.64 (3.06)	19.26 (2.78)	.499	.645	19.7 (3.20)	19.53 (2.91)	0.318	0.75	21.6 (2.52)	19.54 (2.95)	18.5 (3.0)	4.93	0.01^**^	20.4 (2.63)	19.1 (2.87)	19.4 (3.17)	1.419	0.24

^a^parent reported

^*^p < 0.05 ^**^ *p* < 0.01 ^***^ *p* < 0.001

Results of ANOVA tests revealed that the age and education of primary carers were related to children’s PA preference and hours spent in outdoor play (Table 2). Post-hoc tests indicated that children with younger primary carers (20–29 years) were more likely to prefer PA than children with older primary carers (30–39 years and 40+ years) (*F* = 4.93, *p* < 0.01) (parent-reported). Further, parents who had completed tertiary education reported that their children participated in more active play on the weekend (*F* = 3.57, *p* < 0.05) and during the week (*F* = 4.28, p < 0.01) than parents who had completed high school or a technical/trade certificate as their highest level of education (Table 2).

### Physical activity parenting practices and children’s screen-time and active outdoor play (parent-reported)

Pearson correlation tests revealed that a number of controlling and supportive parenting practices were correlated with children’s PA and screen time participation (parent-reported) (Table 3). A child’s exposure to television (*r* = −.191, *p* < 0.05), explicit parental modelling and enjoyment of screen time (*r* = −.345, *p* < 0.001), the use of screen time to reward/control child behaviour (*r* = −.229, p < 0.05) and limiting outdoor play due to weather (*r* = −.229, *p* < 0.01) were negatively associated with children’s parent-reported outdoor active play during the week. Whereas, support/reinforcement from other adults for active play was positively associated with parental reporting of their children’s total hours spent in active outdoor play on weekends and during the week (*r* = .234, p < 0.01). Rules around outdoor play (*r* = 0.195, *r* = 0.12, *p* < 0.05), exposure to television, explicit modelling and enjoyment of screen time (*r* = .450, *r* = .44, *p* < 0.001) were positively associated with parental reporting of their children’s weekend and weekday hours spent engaging in screen time respectively. Support/reinforcement from other adults for active play (*r* = −.303 < 0.001), parent’s perceived importance and value for PA (*r* = −.198, < 0.01), instrumental support for sport (*r* = **−.**208 < 0.01) and explicit modelling and enjoyment of PA (*r* = **−.**342 < 0.001) were negatively associated with parental reporting of their children’s total hours of weekend screen time. Parental limiting or monitoring screen time was negatively associated with parental reporting of their children’s weekend and weekday screen time hours (*r* = −.49 < 0.001) (Table 3).

**Table 3:** Correlations between physical activity parenting practices and children’s physical activity preference, knowledge, screen-time and outdoor play

Variable	Child iPad activity		Parental report	Parental report hours outside play			Parental report hours screen-time		
	Child PA preference score (Pre-FPQ)	Child PA knowledge Score (Pre-FPQ)	Child PA preference (parent Questionnaire)	last 5 weekday	last weekend	Total hours	Last 5 weekdays	Last weekend	Total hours
Controlling factors									
Rules around active play indoors	−0.092	− 0.123	− 0.020	− 0.020	− 0.071	− 0.06	−0.032	− 0.076	−0.05
Rules around active play outdoors	−0.179^*^	− 0.094	− 0.287^***^	−0.159	− 0.063	−0.12	0.12^*^	0.195^*^	.28^***^
Use of PA to reward/control behaviour	0.034	0.025	0.165	0.031	0.122	0.012	−0.11	− 0.104	− 0.08
Limiting or monitoring screen-time	− 0.078	0.058	0.021	0.033	−0.125	−0.05	−.49^***^	−.49^***^	−.49^***^
Limiting outdoor play due to weather	−0.026	− 0.050	− 0.060	−.229^**^	−0.141	− 0.145	0.08	0.059	0.13
Use of screen-time to reward/control child behaviour	−0.026	− 0.050	− 0.060^*^	−.229^*^	− 0.141	− 0.145	0.08	0.059	0.13
Promoting factors									
Explicit modelling and enjoyment of PA	−0.077	0.050	0.228^**^	0.165	0.096	0.166	−.16	−.342^***^	−.26^**^
Verbal Encouragement	0.020	0.096	0.074	0.124	0.081	0.129	−0.02	− 0.099	− 0.13
Instrumental support for sport	− 0.070	0.057	0.119	0.093	0.070	0.100	−0.12	−.208^**^	−.21^**^
Instrumental support for active play	−0.068	−0.007	0.211^**^	0.24^**^	0.27^*^	.220^*^	0.06	−0.131	−0.01
Importance and value physical activity	− 0.052	0.021	.301^***^	−0.041	0.017	−0.023	− 0.12	−.198^**^	−.24^**^
Support/reinforcement from other adults	0.020	0.064	.233^***^	.234^**^	.198^**^	.260^**^	−.230^**^	−.303^***^	−.23^**^
Exposure to TV	0.035	−0.155	−0.027	−.191^*^	0.011	−0.138	0.44^***^	0.450^***^	.57^***^
Explicit modelling and enjoyment of screen-time	−0.389	− 0.041	− 0.170	−.345^***^	0.154	−.33^***^	0.30^***^	0.411^***^	.40^***^
Dependent variables									
Child PA preference (Pre-FPQ)	–	–	–	0.047	.240^**^	0.135	−0.09	0.01	−0.04
Child PA knowledge (Pre-FPQ)	0.096	–	–	0.101	0.099	0.119	0.06	0.01	0.06
Child PA preference (parental questionnaire)	0.029	0.097	–	0.174	0.117	.180^*^	−0.02	−.214^**^	− 0.15

^*^p < 0.05 ^**^ *p* < 0.01 ^***^ *p* < 0.001

### Physical activity parenting practices and children’s physical activity knowledge, preference and behaviour

Pearson correlation tests indicated that children’s knowledge of PA was not correlated with any PA parenting practices (presented in Table 3). Children’s PA preference (Pre-FPQ) was found to be negatively correlated with parental rules around active play outdoors (*r* = **−** 0.179, *p* < 0.05) and positively correlated with parental report of the total hours children spent engaged in outdoor play on the weekend (*r* = **.**240, *p* < 0.01). Child PA preference (parent questionnaire) was found to be negatively correlated with parental report of their children’s weekend screen time (*r* = −**.**214, *p* < 0.01), parental rules around active play outdoors (*r* = − 0.287, *p* < 0.001) and parental use of screen time to reward/control child behaviour (*r* = − 0.06, *p* < 0.05). Child PA preference (parent questionnaire) was also found to be positively correlated with parent’s instrumental for active play (*r* = .211, p < 0.01), parent’s perceived importance and value for PA (*r* = .301, p < 0.001), support/reinforcement from other adults (*r* = **.**233, p < 0.001) and parental report of their children’s total hours spent engaged in outdoor play (*r* = .180, < 0.01).

Multiple regression modeling was used to examine the contribution of key physical activity parenting practices on children’s physical activity preferences (parental questionnaire)(Table 4). Parent-reported child-preferences were selected for this regression as this variable was related to six parenting practices. Key socio-demographic variables were entered at step 1, explaining 7.9% of the total variance in children’s PA preference scores (R^2^ = 0.079) (step 1). Overall, parental age was the only demographic factor which made a significant contribution in explaining children’s PA preference scores (*p* = 0.05). All controlling and supportive parental physical activity practices which were found to significantly correlate with children’s physical activity preferences were added at step 2, explaining an additional 15.7% of the variance in children’s physical activity preference scores, after controlling for demographic factors (R^2^ change = .157, *p* = 0.01). Only rules around active play outdoors (*p* < 0.01) and the use of screen-time to reward/control child behaviour (*p* < 0.05) made significant contributions to changes in children physical activity preferences, thus making the largest contribution to children’s physical activity preferences. The overall model contributed 23.6% (R2 0.236) of the variance in children’s physical activity preferences.

**Table 4:** Hierarchical Multiple Regression: Factors relating to children’s physical activity preferences (parent reported)

Predictor Variable	B	SE	Beta	t	p	R^2^	R^2^ Change
Step 1						0.079	.079^*^
Child gender	−.159	.549	−.026	−.290	.773		
Ethnicity	−1.04	.877	−.108	−1.19	.235		
Parent age	−1.48	.512	−.260	−2.89	.005		
Household income	−.221	.277	−.077	−.796	.428		
Step 2						.236	.157^***^
Rules around active play outdoors	−.220	.083	−.248	−2.64	.009^**^		
Use of screen-time to reward/control child behaviour	.353	.168	.198	2.098	.038^*^		
Explicit modelling and enjoyment of PA	.046	.045	.099	1.024	.308		
Instrumental support for active play	.125	.070	.162	1.80	0.075		
Support/reinforcement from other adults	.183	.143	.121	1.28	.203		
Instrumental support for sport	.112	.109	.093	1.03	.305		

^*^p < 0.05 ^**^ *p* < 0.01 ^***^ *p* < 0.001

## Discussion

The purpose of this study was to examine the relationship between physical activity parenting practices and preschool children’s physical activity knowledge, preferences and child PA and sedentary behaviours (parent-reported). Rather unexpectedly, children’s physical activity knowledge was not found to be correlated with physical activity parenting practices or parental report of their children’s activity behaviours. On the other hand, children’s preferences for physical activity was correlated with a number of demographic characteristics and physical activity parenting practices, with the most influential being parental rules around active play outdoors and parental use of screen-time to reward/control child behaviour. Findings build on existing qualitative literature by providing quantitative evidence of the relationship between children’s PA preferences (child and parent reported) and parental rules around active play outdoors and parental use of screen-time to reward/control child behaviour. Findings will be discussed in the context of existing literature.

### Demographic characteristics and children’s physical activity knowledge and preferences

The demographic factors of child gender, ethnicity and parental age were correlated with children’s physical activity preferences (parent and child reported) and/or physical activity and sedentary behaviours. Children of older parents were more likely to prefer sedentary activities. Literature has presented mixed findings regarding this link [6], nonetheless, a study conducted by Zecevic and others (2010) reported parental age as a negative correlate of children’s physical activity [23]. Follow up analyses in the current study revealed that parents between the ages of 30–39 years participated in significantly less activity than those of 20–29 years and 40–49 years. As 30–39-year-old parents represented 65% of the study population, it could be suggested that the reduced activity levels of this age group may contribute to children’s preference for more sedentary activities. Future research should seek to determine why parents within this age group engage in lower levels of activity, and how underlying factors influence children’s activity preferences.

Boys were more likely to prefer being physically active than girls. This aligns with existing research conducted with primary school children [24], which found that boys had a significantly higher preference for cycling, playing sport at a club and playing outside. Caucasian children and children of parents with a higher level of education were found to prefer more active activities and engaged in more outdoor play during the week. The strength of the associations and the presence of an association between ethnicity and preschool children’s physical activity has varied between studies and remains inconclusive [6, 25]. Ethnic differences in PA preference and participation may be explained by differences in socioeconomic status by ethnic group or the questionnaire used may have not been appropriate for differing ethnicities [25, 26]. Future studies should seek to determine the mechanisms related to ethnicity that underpin differences in physical activity preferences, for example, whether differences in physical activity habits by ethnicity could be attributable to socioeconomic status [21].

There is conflicting literature that both supports and disproves the relationship between parental education and children’s physical activity levels [6, 23, 25, 26]. In the current study, children of parents with a higher level of education engaged in more active outdoor play. As household income was not associated with child activity preference or behaviours in the current study, more research needs to be done to determine how parental education relates to child activity preferences and behaviours.

### Physical activity knowledge, preference and child sedentary and active behaviours (parent reported)

In the current study, children’s knowledge of physical activity was not found to be correlated with children’s active and sedentary behaviour (parent-reported) or any demographic factors. This may be due to high levels of physical activity knowledge in study participants, due to participants being drawn from the educational setting of the childcare centre. Nonetheless this contradicts existing literature which suggests children with increased knowledge of the importance of physical activity are more likely to engage in active play [27]. Children who preferred to be physically active were more likely to engage in physical activity on the weekend and were less likely to engage in screen-time on the weekend. Although existing literature highlights the importance of preschool children’s preference for physical activity as a predictor of activity behaviour, this has been based predominantly on the findings of qualitative literature [3, 5]. The current study adds to existing literature by quantitatively supporting the link between PA child preferences and activity behaviours. The reason for the lack of correlation between children’s activity preferences and weekday activity may be due to the fact that children are at childcare during the week, and thus, have limited control over the activities they engage in [5]. Further, parent’s may be unaware of the exact activity behaviours children engage in during the day while they are at childcare.

### Physical activity parenting practices and children’s physical activity preferences (parent-reported)

There were a number of controlling and promoting physical activity parenting practices that were found to be correlated with preschool children’s activity preferences. After controlling for key demographic factors and parental physical activity practices, only rules around active play outdoors and the use of screen-time to reward/control child behaviour made significant contributions to changes in children physical activity preferences (parent-reported). The PA parenting practice of ‘rules around active play outdoors’ included questions such as ‘do you ask your child not to run outside?’ and ‘ do you ask your child to calm down his/her outdoor play?’. Existing literature supports the link that parent’s attitudes about risky play influence children’s engagement in physical activity, but the current study demonstrates how restrictive rules around outdoor play in the home may also influence children’s activity preferences (parent-reported) [9, 28, 29]. Alternatively, this relationship is likely bidirectional [7], for example, parenting practices may be influenced by child temperament and behavior. This highlights the importance of conducting longitudinal research to gain insight into the bidirectional nature of parent-child relations with regard to activity-related preferences and behaviour. This understanding would assist in determining the target participants of physical activity interventions, for example, parents or children.

Most young children seek out and enjoy challenging play [29]. There is much argument that challenging play affords children a number of benefits including providing them the opportunity to test their limits, explore boundaries and learn to make decisions about injury and risk [9]. Parental rules around outdoor play are arguably fueled by safety concerns and the increasingly regulated and controlled tendencies of western society [14, 15, 18, 29]. Niehues and colleagues (2015) stated that at the root of parenting rules is parental concern of being labelled as ‘incompetent parents’ or having their parenting skills questioned. There are contradictory discourses emerging in literature which suggests that parents understand the importance of encouraging and facilitating their child’s risky play [9, 14], and value their own childhood experiences of themselves engaging in unstructured, unsupervised play [29]. Yet, at the same time, parents consider themselves to be ‘socially assigned’ to make their child’s safety their primary concern and to prevent their child from being injured [28, 29]. Managing these competing societal values along with the staggering amount of information about ideal parenting, makes it understandably difficult for parents to know how to approach this issue. Nevertheless, parents have reported that safety often prevails as a priority over offering children the adventure of age-appropriate risk taking activities [28–30].

This is further complicated by parent’s increasingly busy schedules and dual-working households [14]. Busy parents report that it takes time and additional effort to support autonomy, and that is often easier to take the safer option and say ‘no’ to riskier activities [3, 30]. This may contribute to children engaging in more screen-time, which parent’s may consider as low risk. This theory is supported by the current study as findings indicate that the more parents employ restrictive rules around outdoor play, the less likely their children was to prefer active outdoor play and the more likely they were to engage in screen-time.

Another influential contributor to children activity preferences was parent’s use of screen-time to reward and control their child’s behaviour. This PA parenting practice included questions such as ‘Do you offer screen-time as a reward for good behaviours?’ and ‘Do you take away screen-time as a punishment for bad behaviour?’. Qualitative literature suggests that screen devices are being more commonly used as a reward or punishment or conflict reduction [31]. In the current study, parent use of screen-time to reward/control child behaviour led to children preferring more sedentary activities and was also negatively correlated with children’s active outdoor play. This is consistent with existing literature which suggests that this parenting practice to manage children’s behaviour leads to an increase in children’s screen-time [8, 32].

There is limited existing research which explains the correlation between children activity preferences and parent’s use of screen-time to reward and control their child’s behaviour. However, it has been suggested that rewarding or encouraging behaviours with screen-time further elevates the status of screen-time by using it as a tool to drive and shape their behaviour. It can also be hypothesized that by using screen-time as a reward or punishment, encourages children to think of screens as a recreational, exciting activity as opposed to a more functional tool. Nevertheless, several studies have contradicted this finding, and suggest that adult rules on screen use can effectively deter children from participating in excessive TV viewing and computer use, if supported concurrently with adult modelling of low screen use [33]. The current study did not find that parental explicit modelling and enjoyment of screen-time was correlated with children's activity preferences. Finally, as the regression model accounted for 23.6% of the variance in children’s activity preferences, further research is needed to explore variables that were not accounted for in the current research which may be influencing children’s activity preferences.

## Limitations

This study is subject to limitations. First, the researchers recruited participants from early childcare educational settings. This limits the generalizability of findings, as children attending childcare may be more educated regarding the importance of physical activity. Second, children attending these centres, particularly in metropolitan areas can often be of a higher sociodemographic background e.g. median income in survey was AUD$90,000 and the median income in Australia was AUD$75,000 [34]. Last, while the instrument used has been shown to be reliable in previous studies to gather parental report of children’s activity and sedentary behaviours, social desirability bias could have resulted in a mis-estimation of the relationship between parenting practices and children’s activity preferences or behaviours [35].

## Conclusions

This study identified that parenting practices are not only associated with children’s parent-reported active and sedentary behaviours, but also how children prefer to play. Children’s activity preferences for physical activity (parent-reported) was found to be correlated with parental rules around active play outdoors and parental use of screen-time to reward/control child behaviour. Given the link between children’s activity preferences and behaviours in early childhood, contextual factors shaping these parenting practices warrant consideration.

Competing societal values of the importance of encouraging children’s risky play and the need to prevent children from being injured, coupled with parent’s busy schedules and the abundance of parenting advice they receive, may be leading to parents feeling ambivalent of how to promote active play for their children. Future public health practice needs to consider this confusion and find ways to assist parents to navigate the information being provided to them. Further, future research should seek to clarify the relationship between children’s activity preferences and parent’s use of screen-time to reward and control their child’s behaviour, given the developmental and behavioural health risks associated with excessive media/screen exposure in early childhood. Finally, given the inconsistencies in the literature regarding the relationships between the demographic factors of parental age, education, child gender, ethnicity and children’s activity preferences, future research should consider what could be underpinning these relationships in different contexts. This may help to identify those that could benefit from assistance regarding how to encourage their child’s active play or, assist in developing interventions with broader benefits.

## Data Availability

Interested researchers (who meet criteria for access to confidential data) may contact the corresponding author of our manuscript for access to the datasets generated during and/or analysed during the current study.

## Abbreviations

AUD: Australian dollars
n: Number of participants
PA: Physical activity
SD: Standard deviation
